# mTOR hyperactivation in Down Syndrome underlies deficits in autophagy induction, autophagosome formation, and mitophagy

**DOI:** 10.1038/s41419-019-1752-5

**Published:** 2019-07-22

**Authors:** Matteo Bordi, Sandipkumar Darji, Yutaka Sato, Marian Mellén, Martin J. Berg, Asok Kumar, Ying Jiang, Ralph A. Nixon

**Affiliations:** 1 Center for Dementia Research, Nathan Kline Institute, Orangeburg, NY USA; 2 Department of Psychiatry, New York University Langone Medical Center, New York, NY USA; 3 Department of Biology, University of Rome Tor Vergata, Rome, Italy; 4 Experimental Sciences, Universidad Francisco de Vitoria, Pozuelo de Alarcón, Madrid, Spain; 5 Department of Cell Biology, New York University Langone Medical Center, New York, NY USA; 6 NYU Neuroscience Institute, New York University Langone Medical Center, New York, NY USA

**Keywords:** Autophagy, Mitophagy, Molecular biology, Transcriptomics

## Abstract

Down syndrome (DS), a complex genetic disorder caused by chromosome 21 trisomy, is associated with mitochondrial dysfunction leading to the accumulation of damaged mitochondria. Here we report that mitophagy, a form of selective autophagy activated to clear damaged mitochondria is deficient in primary human fibroblasts derived from individuals with DS leading to accumulation of damaged mitochondria with consequent increases in oxidative stress. We identified two molecular bases for this mitophagy deficiency: PINK1/PARKIN impairment and abnormal suppression of macroautophagy. First, strongly downregulated PARKIN and the mitophagic adaptor protein SQSTM1/p62 delays PINK1 activation to impair mitophagy induction after mitochondrial depolarization by CCCP or antimycin A plus oligomycin. Secondly, mTOR is strongly hyper-activated, which globally suppresses macroautophagy induction and the transcriptional expression of proteins critical for autophagosome formation such as ATG7, ATG3 and FOXO1. Notably, inhibition of mTOR complex 1 (mTORC1) and complex 2 (mTORC2) using AZD8055 (AZD) restores autophagy flux, PARKIN/PINK initiation of mitophagy, and the clearance of damaged mitochondria by mitophagy. These results recommend mTORC1-mTORC2 inhibition as a promising candidate therapeutic strategy for Down Syndrome.

## Introduction

Down syndrome (DS, HSA21), among the most common genetic disorders, is caused by a total or partial trisomy of chromosome 21^1^. Individuals with DS display a considerable range of symptoms, including neurocognitive disabilities of variable severity, due to the high genetic complexity and adaptive mechanisms that partially offset the presence of an extra copy of HSA21^2^. Additionally, DS patients invariably develop Alzheimer’s disease (AD) neuropathobiology by the fifth decade of life^3^ with dementia onset by the age of 65. AD emergence in DS is likely attributable to the aneuploidy of the *APP* gene although the involvement of other genes localized on Chr 21, such as *RCAN1* and *DYRK1A*, cannot be excluded^4^. Mitochondrial anomalies produce deficits in DS brain and other tissues, including impaired ATP production, oxidative stress and increased vulnerability to cell death^5–9^. Similar mitochondrial abnormalities develop in mouse models of DS, including Ts1 lacking an extra *APP* allele^10^.

Studies have shown that mitochondrial damage triggers their rapid elimination by macroautophagy^11^, prompting us to consider whether macroautophagy deficits form a basis for the abnormal mitochondrial phenotype and its pathological consequences. Macroautophagy (hereafter referred to as autophagy) is a major lysosomal degradative pathway for the elimination and recycling of obsolete and damaged cytoplasmic components and turnover of vesicular organelles, including mitochondria^11,12^. Compelling genetic and pathological evidence has implicated autophagy dysfunction in the pathogenesis of multiple neurodegenerative diseases, including AD^13,14^.

Various forms of selective autophagy have been identified, including the removal of damaged mitochondria (mitophagy)^15^. Mitophagy is initiated by signaling events following mitochondrial membrane depolarization leading to the recruitment of specific adaptors including PINK1–PARKIN representing the most well-characterized pathway. Herein mitophagy is activated with PINK1 accumulation on the mitochondrial outer membrane upon mitochondrial depolarization, then phosphorylating ubiquitin promoting translocation of the E3 ubiquitin ligase PARKIN (*PARK2*)^16,17^. PARKIN builds ubiquitin chains on mitochondrial external membrane proteins recruiting specific autophagic cargo receptors, such as p62 (*SQSTM1*) and NDP52, facilitating sequestration into the autophagosome (APs)^17,18^. Moving forward, mitophagy and bulk autophagy employ similar sequestration and clearance steps; however, the differential regulation of these two processes is not fully understood.

The mTOR complex1 (mTORC1), a master regulatory protein kinase^19^, negatively regulates autophagy by down-regulating the ULK1 complex^20^. mTORC1 itself is positively regulated by the small GTPase Rheb on lysosomes^21^, and by the PI3K/AKT cascade downstream of receptor tyrosine kinases^22^. Inhibiting mTORC1 with activation of ULK1 complex stimulates downstream components of the autophagic pathway, such as PI3K-cIII complex and ATG-related proteins, resulting in nucleation, elongation and formation of APs^23^. ATGs -7, -3, and -5 are essential proteins for the correct formation of APs, are involved in two ubiquitin-like systems and binding of LC3/GABARAPs with phosphatidylethanolamine (PE)^24^. Interestingly, studies of DS frontal cortex and hippocampus report aberrant hyperactivation of the AKT/mTOR signaling pathway in DS brain^25,26^ suggesting imbalances in autophagy flux regulation in DS leads to negative effects on mitochondrial turnover.

Here, we establish mitophagy impairment in DS fibroblasts and identify two underlying molecular mechanisms. First, abnormally lowered levels of PARKIN coupled to altered PINK1 activation induce mitochondrial depolarization. Secondly, and more significantly in this model is mitophagy suppression linked to down-regulated rate of autophagy caused by mTOR hyper activity and accompanying reduction in expression of key ATG proteins. We further demonstrate that mitophagy and overall autophagy can be rescued by inhibiting mTORC1 and mTORC2 with AZD8055, a novel inhibitor of mTOR kinase activity^27^, suggesting innovative avenues for treatment of DS.

## Materials and methods

### Cell culture and transfections

Human forearm skin fibroblasts from DS and diploid (2 N) age-matched controls (5 months, 2 and 5 years old) from the Coriell Cell Repositories (AG06922, AG07095, AG07096, GM08680, GM05381, AG04823) were cultured in DMEM medium (Life Technologies #11995-065) containing 10% fetal bovine serum (Life Technologies #10270), 100 mg/L sodium pyruvate, 100 units/mL Penicillin-Streptomycin (Life Technologies #15140-122) at 37 °C in a 5% CO2 atmosphere. Cell passage number ranged from p7 to p12, and cells at 85–90% confluency were used throughout the study. Fibroblasts were seeded on glass coverslips in 12-well dishes for immunolabeling or 100–150 mm dishes for biochemical applications as follows.

### Cell treatment

As lysosomal-inhibiting agents, we used the following: (a) 10 μM Leupeptin inhibitor of cysteinyl and serine proteases) and Pepstatin A (aspartyl proteases)(Peptide Institute, Japan) for 24 h; (b) 10 nM Bafilomycin A (Bafil), a specific inhibitor of the lysosomal vacuolar type H^+^-ATPase (V-ATPase) that blocks lysosomal degradation^28^ (Sigma B1793) for 6 h; (c) 50 nM Concanamycin A (ConA), a second potent V-ATPase inhibitor^29^ (Sigma 27689) for 2 h. For mitochondria depletion via induced mitochondrial membrane depolarization, cells were treated with 20 μM CCCP^15^ (Sigma C2759) for 6 h or with a combination of 1 μM antimycin A and 1 μM oligomycin (AA/OA) inhibiting the electron transport chain complex III and of ATP synthase, respectively^28^ (Sigma A8674/O4876) for 1, 3, 6 and 24 h. Cells were treated with 0.1 μM AZD8055 (AZD), a recently introduced ATP-competitive inhibitor directly targeting the mTOR catalytic site, blocking both mTORC1 and mTORC2^30^ (Selleckchem) for 2, 4 and 8 h as reported in figure legends.

### RNA sequencing (RNA-Seq)

RNA was extracted from human fibroblasts using RNeasy Micro Kit (Qiagen) according to the manufacturer’s specifications and digested with DNAse I (Sigma). RNA quantity and quality were determined with a Nanodrop 1000 spectrophotometer (Wilmington, DE) and Agilent 2100 Bioanalyzer system. Three biological replicas were produced per each cell line. 500 ng of total RNA were prepared using TruSeq RNA Sample prep kit V2 (Ilumina Inc., San Diego, CA, USA) following manufacturers’ instructions for further sequencing in HiSeq 2000 (Ilumina Inc.). Quality of libraries was assessed using an Agilent 2100 Bioanalyzer system. We obtained on average 52 million 50 bp single-end reads and 50 bp paired-end reads per sample that were separately aligned to the human genome (hg19, GRCh37) downloaded from University of California Santa Cruz Genomics Institute. STAR software (version 2.3.1z) was used for processing reads with the default parameters. The resulting aligned data in bam format was assembled into transcripts using HTSeq (version 0.6.1p2).

### RNA-Seq data analyses

To increase the degree of freedom and power of statistical analysis, additional human skin fibroblast samples from age matched Trisomy 21 and normal unrelated individuals from ref. ^31^. Samples GM04616, AG06922, GM08447, GM05756, and GM00969 were used in conjunction with our samples to make a final number of 4 DS fibroblast lines and 5 2n lines. With biological replicates, this count rises to 8 and 9, respectively. The data files have been deposited in the Gene Expression Omnibus (GEO) database under the accession number GSE126910. The entire analysis was performed in R. For differential gene expression analysis, EdgeR and Limma^32^ packages from R/Bioconductor were used. The correlation between biological replicates (since they were from the same patients) was estimated and this inter-subject correlation was fitted into the linear model to avoid an overfit and inflation of p-values (i.e., reducing the false positives). Counts per million (CPM) have been used after TMM- normalization in the texts and figures. Gene Set Enrichment Analysis (GSEA) of gene sets within the expression data was carried out using mroast functionality from ROAST^33^, which assesses the overall magnitude and direction of change of a gene set within a comparison in the whole expression profile. Gene sets were considered to be significantly enriched if the adjusted (Benjamini–Hochberg) *p*-value, i.e., FDR (*q*-value) was <0.01 in pairwise comparisons using 100,000 random rotation. ROAST were used on the mTOR targets list, which was obtained from those genes described to be upregulated upon constitutive hyperactivation of mTOR caused by TSC1 KO;^34^ the list of transcriptional factors described to be essential for supporting mitophagy activation and the list of transcriptional factors regulating mitochondrial biogenesis are described in Supplementary Table 1.

### Preparation of cDNA and qPCR

cDNA was prepared from total RNA using TaqMan Reverse Transcription Reagent kit N808-0234 (ThermoFisher/Life Technologies) according to manufacturer’s instructions and using Random hexamers. Following reverse transcription, 20 ng sample cDNA were loaded in triplicate into wells of a 96-well optical reaction plate containing appropriate target gene primer (see below; Taq-Man validated primers purchased from Life Technologies/ThermoFisher) in a total reaction volume of 20 μl. β-ACTIN (ACTB) was used as a housekeeping gene. qPCR was performed in the ABI Prism 7900HT Sequence Detection System (Applied Biosystems Branchburg, NJ) as described previously^14^. We note here that although PARK2 (PARKIN) levels were negligible via RNA-Seq, qPCR was sufficient to detect and quantify as described in Results. Following qPCR, the target genes were normalized against the housekeeping gene. Results were calculated using the ΔΔCt method (Applied Biosystems, Branchburg, NJ Bulletin #2). Sample values were recalculated and expressed as percent control. Outliers were recognized as values falling beyond two standard deviations of the mean and were discarded from the subsequent analyses.

List of primers used:

Hs01060665_g1*ACTB*

Hs00197348_m1*ATG7*

Hs00157205_m1*CTSD*

Hs01054576_m1*FOXO1*

Hs01038325_m1*PARK2*

Hs00260868_m1*PINK1*

Hs01061917_g1*SQSTM1*

Hs00177504_m1*ULK1*

### Immunofluorescence

Fibroblasts were seeded into a 12-well plate with coverslip and grown to 40–60% confluence before treatment. Following incubation cells were washed with PBS and fixed with 4% paraformaldehyde at room temperature for 15 min. After permeabilization with 100 μg/mL Digitonin (Sigma D141) in PBS for 15 min, cells were incubated overnight at 4 °C with LC3 (#M152-3, MBL for ICC) and with TOM20 (#sc-11415, Santa Cruz Biotechnology) in PBS with 3% normal goat serum (v:v). Cells were then washed in PBS and incubated for 1 h with fluorescent conjugated secondary antibodies (ThermoFisher/Life Technologies). Mitochondrial ROS production was evaluated using MitoSOXTM (3 µM, Molecular Probes) and mitophagy was evaluated using a combination of LysoTracker Red DND-99 (100 nM; L-7528) with Mitotracker Green FM (100 nM; M-7514) according to the manufacturer’s instructions (ThermoFisher). Immunofluorescent images for LC3 and TOM20, for MitoSox staining or double labeling with Mitotracker Green and Lysotracker Red were collected on a confocal microscope (Zeiss LSM510). Co-localization and quantification of LC3 puncta per cell were determined using the JACoP Plug-In on ImageJ (NIH) software. Fluorescent images were adjusted for brightness, contrast and color balance using Adobe Photoshop CS (San Jose, CA, USA).

### Mitochondrial mass

Cells were seeded in 96 microplate F-bottom (Greiner Bio-One International #655090) and incubated with MitoTracker Green FM as described above. Fluorescence was measured at 488 nm using a SpectraMax M5 plate reader (Molecular Devices).

### SDS-PAGE and Western blotting

Following treatment cultures were rinsed with ice-cold PBS, and lysed in RIPA buffer (50 mM Tris-HCl pH 7.4, 1% Triton X-100, 150 mM NaCl, 0.25% sodium deoxycholate, 0.1% SDS, 1 mM EDTA, 5 mM MgCl_2_) containing protease inhibitor cocktail (Sigma, St. Louis, MO, USA) and phosphatase inhibitors (20 mM NaF, 10 mM β-glycerophosphate, 1 mM Na_3_VO_4_) via sonication and incubation in ice for 30 min. A clear supernatant was obtained by centrifugation of lysates at 15000 rpm for 15 min. Mitochondrial/cytosolic fractionation was performed as described previously^35^. Cells were harvested in hypotonic buffer (15 mM MgCl_2_, 10 mM KCl, 10 mM Tris-MOPS, pH 7.4), supplemented with protease inhibitor cocktail and incubated for 15 min in ice. 1 Volume of 2x Mitobuffer (400 mM sucrose, 10 mM TES, pH 7.2, 100 mM EGTA, 2 μM DTT) was added and the cell suspension was homogenized for 40 strokes with a Dounce homogenizer. Samples were centrifuged twice at 900 × *g* for 10 min at 4 °C to eliminate cell nuclei and unbroken cells. The resulting supernatant was centrifuged at 12,000 × *g* for 15 min at 4 °C to recover the heavy-membrane pellet enriched in mitochondria, and the resulting supernatant was stored as cytosolic fraction. Protein concentration was determined using the BCA protein assay (Thermo Scientific #23225); and samples subjected to SDS-polyacrylamide gel electrophoresis followed by Western blotting described previously^14^. Following processing, nitrocellulose membranes (Pall) were subjected to enhanced chemiluminescent substrates (Invitrogen, WP20005 or Millipore WBKL50500) and exposed to X-ray film (Ewen-Parker, RX-B); bands were quantified using ImageJ (NIH) software. Target proteins were normalized against β-ACTIN or VDAC1, unless otherwise noted.

Antibodies for Western analyses: AKT (#4685), phospho473-AKT (#4060), phospho308-AKT (#2965), ATG3 (#3415), ATG7 (#2631), COXIV (#4850), FOXO1 (#2880), H3 (#4499), mTOR (#2983), NDP52 (#60732) phospho2448-mTOR (#2971), P70S6K (#2708), phospho389-P70S6K (#9234), TFEB (#4240), ULK-1 (#4773), phospho758-ULK1 (#6888) and VDAC1 (#4866) from Cell Signaling; p62/SQSTM1 (610832) from MBL; LC3B (NB100-2220) and PINK1 (BC100-494) from Novus Biologics; PARKIN (Ab77924) and OPTINEURIN (Ab23666) from Abcam; TOM20 (#sc-11415) from Santa Cruz Biotechnology; βIII-TUBULIN (SAB4700544) from Sigma.

### Carbonylation protein assay

We used the Oxidized Protein Western blot Kit (#ab178020) from Abcam, as per manufacturer’s instructions.

### Electron microscopy

After washing with serum-free medium, cells were fixed with 4% paraformaldehyde/1% glutaraldehyde/5% sucrose in 0.1 M sodium cacodylate buffer, pH 7.2 (Electron Microscopy Sciences, PA) for 24 h at RT. Cells then washed three times with 0.1 M sodium cacodylate buffer, post-fixed in 1% osmium tetroxide and progressively dehydrated in a graded series of ethanols (50–100%) followed by embedding in Epon (Electron Microscopy Sciences) for at least 3 days at RT. Epon-embedded blocks were sectioned and placed on copper grids for structural analysis via Transmission Electron Microscopy (TEM) with magnification ×5800, ×13500 or ×25000. EM images in each condition were randomly selected and captured, and the number of damaged mitochondria was manually counted. Vacuoles within mitochondria were quantified upon AZD8055 treatment after 2 and 8 h and expressed as a ratio of autophagosomes to autolysosomes inside mitochondria/total number of autophagosomes and autolysosomes per field.

## Results

### Damaged mitochondria accumulate in DS fibroblasts in association with oxidative stress

**Fig. 1:**
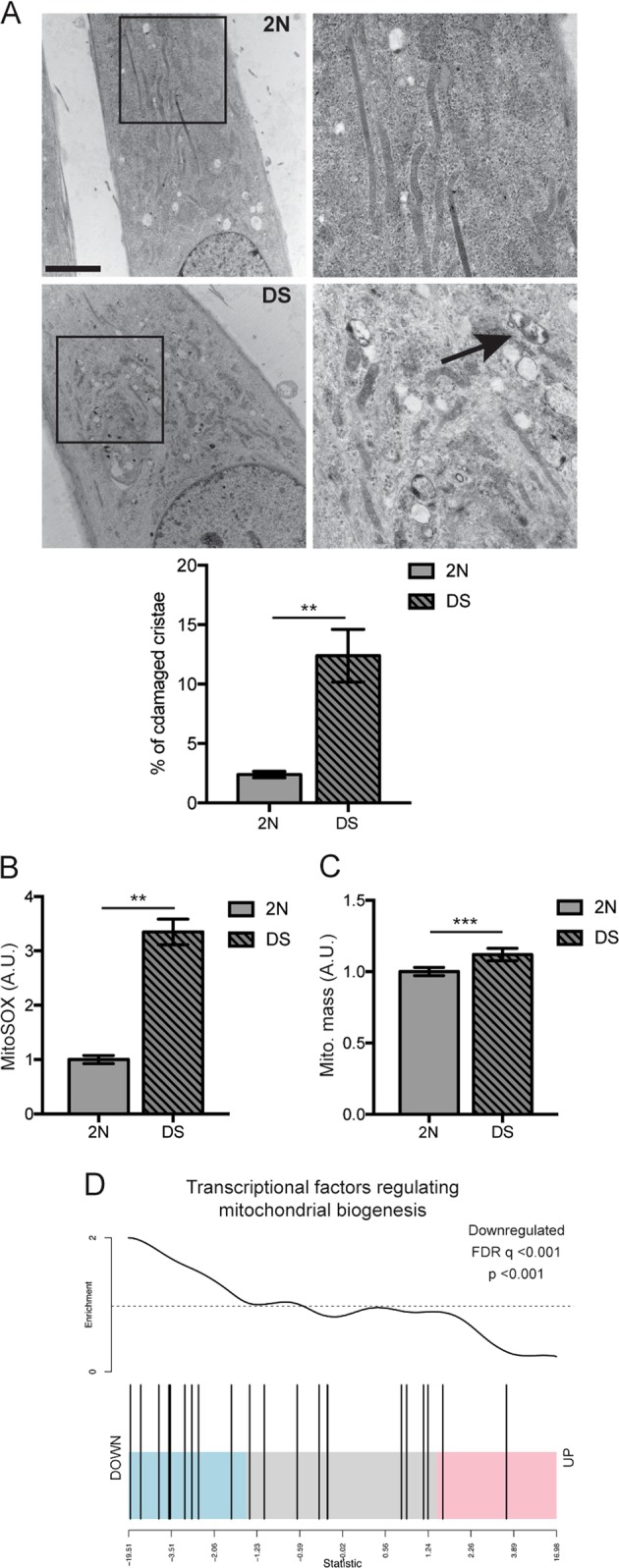
Damaged mitochondria accumulation in DS fibroblasts and resulting increase of oxidative stress. **a** Electron microscopy analysis of 2 N and DS cells; section indicated by the square is shown at higher magnification in the right panels. Black arrow points to example of an abnormal mitochondrion. Quantification shown as percentage of damaged mitochondria in respect to total number. Scale bar 500 nm. **b** Quantification of MitoSOX^TM^ intensity fluorescence reported as fluorescence arbitrary units (A.U.) for three independent experiments. The analysis was done using the software ImageJ. **c** Quantification of mitochondrial mass using Mitotracker green intensity fluorescence, acquired through SpectraMax M5 Microplate Reader and reported as fluorescence arbitrary units (A.U.) from at least three independent experiments. **d** Gene set analysis barcode plot of transcriptomic changes of transcriptional factors (TFs) regulating mitochondrial biogenesis^7,42,43^ (for list of genes, see Supp. Table 1) in DS fibroblasts compared to 2 N fibroblasts. The vertical bars represent TFs (genes) and are ranked horizontally by moderated t-statistics (x-axis). Pink, blue and grey shaded rectangles mark upregulated, downregulated and genes that are unchanged, respectively. The black trace above the bars shows relative enrichment of genes using Limma’s ROAST function (see Methods section). The analysis shows significant downregulation of TFs regulating mitochondrial biogenesis in DS compared to 2 N fibroblasts. (*p* < 0.001; FDR < 0.001_).._ Statistical analysis in A, B and C was performed using Student’s *T*-test. (*p < 0.05; **p < 0.01; ***p < 0.001)

To dissect molecular mechanisms contributing to the DS phenotype, we evaluated alterations to the mitochondrial network. We first examined DS and 2 N control fibroblasts by TEM and fluorescent imaging techniques to highlight differences in mitochondrial ultrastructure and function. EM analysis revealed significantly more mitochondria with abnormal morphology in DS fibroblasts compared to 2N (*p* < 0.05; Fig. 1a). Altered cristae organization, such as internal structures collapsed against one side (Fig. 1a) were associated with decreased mitochondrial respiration efficiency and increased reactive oxidative stress (ROS)^36^. Accordingly, DS cells exhibited elevated levels of mitochondrial superoxide (*p* < 0.01; Fig. 1b, Suppl. Fig. 1a), and oxidative carbonylated proteins (Suppl. Fig. 1b) correlating with increased ROS production, confirming earlier studies^7–9,37^. Accordingly, levels of SOD2, the main scavenger of superoxide into the mitochondria^38^, were lowered in DS cells (*p* < 0.01; Suppl. Fig. 1c, d), while VDAC1, a critical regulator of mitochondrial metabolic and energetic functions^39^, was unaltered (Suppl. Fig. 1c, d). Transcriptomic analyses comparing gene expression related to mitochondrial function and ROS revealed reduced *SOD2* expression (Suppl. Fig. 1e) and down-regulation of other important antioxidant enzymes, including TXNRD1/2, (Suppl. Fig. 1e). However mitochondrial fission and fusion genes regulating mitochondrial dynamics^40^ were unchanged (Suppl. Fig. 1g).

Mitochondrial mass was significantly elevated in DS cells compared to 2 N (*p* < 0.001) (Fig. 1c). To support this finding, we performed GSEA^41^ using a gene set of relevant transcriptional factors (TFs) regulating mitochondrial biogenesis, including *PPARGC1A* (PGC-1α), a master regulator of mitochondrial biogenesis^7^, *PPARG* (PPARgamma), *TFAM*, *STAT3*, *TFB1M* and *FOXO1*^42,43^ showing these were significantly downregulated in DS (*p* < 0.001, FDR *q* < 0.001; Fig. 1d and Suppl. Fig. 1f). Hence, despite transcriptional evidence for reduced mitochondrial biogenesis, DS cells exhibit increased mitochondrial mass, an accumulation of damaged mitochondria, and an upsurge of ROS, collectively suggesting that clearance of defective mitochondria may be impaired.

### Impaired mitophagy responses are associated with PINK1/PARKIN dysregulation

**Fig. 2:**
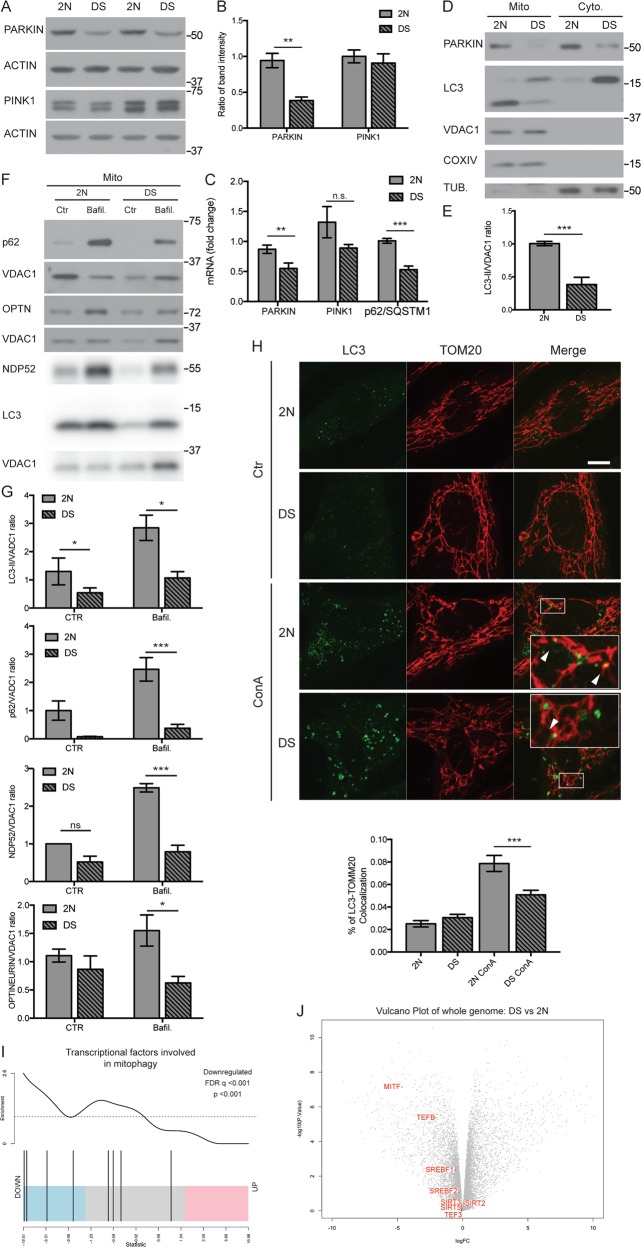
PARKIN down regulation and impairment of mitophagy in DS fibroblasts. **a, b** Whole-cell extracts from 2 N and DS cells were analyzed by western blot for PARKIN and PINK1. Each quantitative data was normalized with ACTIN. (*n* ≥ 3). **c** 2 N and DS cells were assessed for *PARKIN* and *PINK1* mRNA by quantitative real-time PCR. mRNA levels were normalized to *ACTIN* mRNA, used as internal control. Data display the fold-changes of *PARKIN*, *PINK1* and *SQSTM1* (p62) mRNAs relative to control cells (*n* ≥ 3). **d** Mitochondrion/cytosol fractionation was performed to assay the presence of PARKIN and LC3-II within the mitochondrial fraction. VDAC1 and COXIV were used as markers of purity for mitochondrial fraction. βIII-TUBULIN was used as a marker of the cytosolic fraction. **e** Quantitative data of LC3-II in the mitochondrial fraction, normalized with VDAC1 (*n* = 5). **f** Mitochondrial fractionation was performed to assess the accumulation of p62, LC3-II, NDP52 and OPTINEURIN (OPTN) within the mitochondria in untreated and treated fibroblasts with a lysosomal-inhibiting agent, Bafilomycin A (Bafil., 10 nM) for 6 h. Each protein was normalized with VDAC1 with quantitation in (**g**). (*n* ≥ 3) **h** 2 N and DS cells were treated with Concanamycin A (ConA, 50 nM) for 2 h to block lysosome function. The cells were fixed and immunostained for LC3 and TOM20 in order to evaluate mitophagy. White arrows point to the co-localization of puncta into the high magnification image of the boxed area. Scale bar 10 μm. The graph shows the percentage of LC3/TOM20 colocalization calculated by JACoP plugin of ImageJ. Quantitation based on a minimum of 50 cells per conditions from three independent experiments. **i** GSEA analysis of transcriptomic changes comparing DS fibroblasts to 2 N fibroblasts, using a list of transcriptional factors that have been described to be involved in mitophagy^46,47^ (for list of genes, see Supp. Table 1). As described in Fig. 1d, the vertical bars represent genes ranked horizontally by moderated *t*-statistics; Pink, blue and grey shaded rectangles upregulated, downregulated and genes with no change respectively. The plot shows significant downregulation of TFs involved in mitophagy in DS compared to 2 N fibroblasts (*p* < 0.001; FDR < 0.001). **j** Volcano plots for whole transcriptomes of differentially expressed genes between DS and 2 N fibroblasts, with TFs involved in mitophagy, used in (I) for GSEA analysis highlighted with labels. Statistical analysis was performed using Student’s T-test or one-way ANOVA with Tukey’s multiple comparisons test. (**p < 0.01; ***p < 0.001). Immunoblots reported are from one representative experiment

To investigate the basis for abnormal accumulation of damaged mitochondria in DS fibroblasts, we assessed the competence of PINK1-PARKIN dependent mitophagy^15^. Notably basal levels of PARKIN protein were ≈60% lower in DS fibroblasts than in 2 N cells (*p* < 0.001, Fig. 2a, b), concomitant with lowered PARKIN mRNA (≈45%, *p* < 0.01; Fig. 2c). By contrast, PINK1 protein and mRNA levels in DS cells were unchanged (Fig. 2a–c).

We next investigated whether the lowered expression of PARKIN could impair mitophagy. Immunoblot analyses of enriched mitochondrial fractions from DS cells show PARKIN loss was associated with a 60% decrease in LC3-II levels (*p* < 0.001; Fig. 2d, e), suggesting a mitophagy deficit compared to 2 N. To exclude elevated degradation of damaged mitochondria as an explanation, we repeated the analyses in the presence of Bafil, a specific inhibitor of lysosomal acidification^28^. Treatment reduced mitochondrial-LC3-II co-localization in DS cells, together with p62, NDP52 and OPTINEURIN, three cargo receptors central for autophagic clearance of mitochondria^44^, supporting the conclusion that initial stages of mitophagy were impaired (Fig. 2f, g). By RNA-seq analysis, no changes were observed in *CALCOCO2* (NDP52) and *OPTN* (OPTINEURIN) mRNA levels, while *MAP1LC3B* (LC3B) significantly increased between DS and 2 N cells (Suppl. Fig. 1h). *SQSTM1* (p62) showed a trend of decrease that became significant when analyzed by qPCR (*p* < 0.001; Fig. 2c).

To further establish a defect in mitophagy, we assessed colocalization between endogenous LC3 puncta and TOM20, a mitochondrial outer membrane protein (Fig. 2h). Consistent with impaired mitophagy initiation, colocalization decreased in DS cells (*p* < 0.001; Fig. 2h) in the presence of the v-ATPase inhibitor, ConA^29^. Notably, basal colocalization was not significantly lowered in DS cells compared to 2 N cells (Fig. 2h), pointing to lysosomal activity impairment noted previously^45^.

We next evaluated alterations of genes encoding key proteins regulating the mitophagy pathway. GSEA and transcriptomic analysis revealed overall significant downregulation of TFs essential for supporting mitophagy activation^46,47^ in DS (Fig. 2i–j), strengthening links between mitophagy impairment and accumulation of damaged mitochondria.

### Alteration of the PINK1/PARKIN pathway is associated with mitophagy deficits

**Fig. 3:**
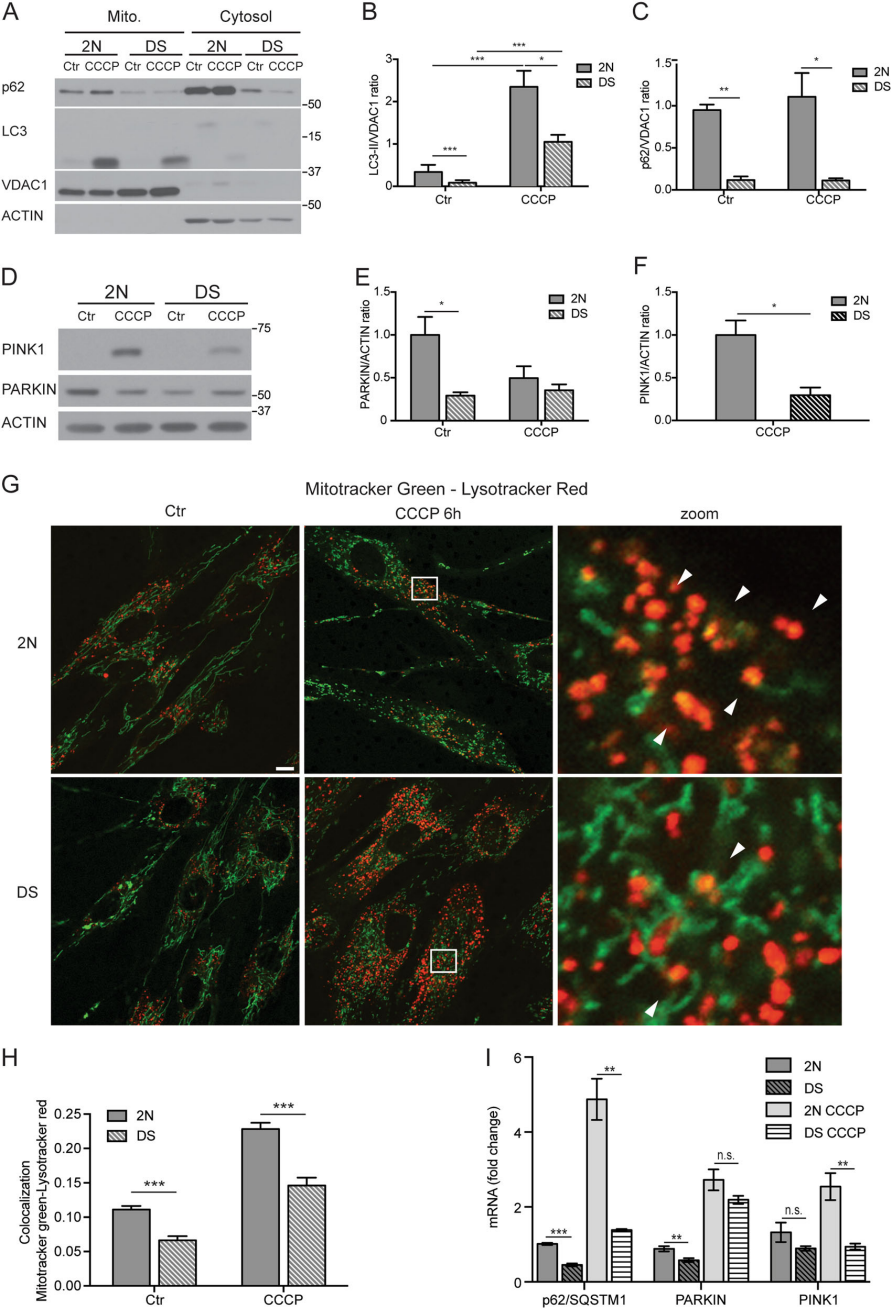
Alteration of PARKIN leads to an impairment of mitophagy upon CCCP treatment. **a** 2 N and DS were treated with CCCP (20 μM) for 6 h and mitochondrion/cytosol fractionation was performed to assay the accumulation of LC3-II and p62 within the mitochondrial fraction. VDAC1 and ACTIN were used as a loading control for mitochondrial and cytosolic fractions, respectively. **b, c** Quantitative data of LC3-II and p62 in the mitochondrial fraction were normalized with VDAC1 (*n* = 3). **d** The cells were treated with CCCP (20 μM) for 6 h and the whole-cell extracts from 2 N and DS were analyzed by western blot for PARKIN and PINK1. **e** Quantification of PARKIN normalized with ACTIN (*n* ≥ 3). **d** Quantification of PINK1 after 6 h of CCCP, each quantitative data was normalized with ACTIN (*n* ≥ 3). **g** Representative confocal images of cells stained with Mitotracker green (mitochondrial marker) and Lysotracker red (lysosomal marker) upon CCCP treatment (20 μM for 6 h). White arrow heads point to the co-localization within puncta from the boxed region at high magnification shown on the right. Scale bar 10 μm. **h** The graph shows the percentage of Mitotracker green /Lysotracker red colocalization calculated by JACoP plugin of ImageJ. Minimum 50 cells per condition were counted from three independent experiments. **i** 2 N and DS cells were treated with CCCP (20 μM) for 6 h and were assessed for *SQSTM1* (p62), *PARKIN* and *PINK1* mRNA by quantitative real-time PCR. mRNA levels were normalized to *ACTIN* mRNA, used as internal control. Data display the fold-changes of *SQSTM1*, *PARKIN* and *PINK1* mRNAs relative to control cells (*n* = 3). Statistical analysis was performed using Student’s *T*-test or one-way ANOVA with Tukey’s multiple comparisons test. (*p < 0.05; **p < 0.01; ***p < 0.001; *n.s.* no significant difference). Immunoblots reported are from one representative experiment

To investigate the role of decreased PARKIN in impairing mitophagy, we activated mitophagy by treating DS and 2 N control fibroblasts cells with CCCP^15^. LC3-II levels were elevated in the mitochondrial fraction in response, albeit significantly lower in DS compared to 2 N (*p* < 0.05; Fig. 3a, b), suggesting impaired mitophagy. WB analysis confirmed this observation wherein total lysates showed 70% lower PINK1 accumulation in DS cells (*p* < 0.05 vs control; Fig. 3d, f). Indeed, upon mitochondrial membrane depolarization, PINK1 is stabilized on the outer membrane where it promotes PARKIN-mediated mitophagy initiation^15^. In human fibroblasts, p62 seems not to be involved in CCCP-induced mitophagy, as also described in HeLa cells^17,48^. Accordingly, p62 changes were minimal in response to CCCP (6 h) in 2 N and DS cells, which nevertheless maintain a modest association with mitochondria irrespective of treatment (Fig. 3a, c).

We then evaluated the delivery of mitochondria to lysosomes by measuring colocalization of Mitotracker green and Lysotracker red. Colocalization of these two markers at baseline and upon CCCP treatment in DS cells (*p* < 0.001; Fig. 3g, h) is consistent with a reduced capacity to eliminate damaged mitochondria compared to 2 N. We identified mitochondrial stress induced by CCCP promoting expression of *PARKIN* and *PINK1* genes^49,50^. Herein *PARKIN* expression via qPCR analysis was similarly upregulated in both cell lines after 6 h of CCCP treatment (Fig. 3i); however, we did not see any rise of PARKIN protein levels in DS cells, but significant lowering in 2 N (Fig. 3d, e), as noted also by Rakovic and coauthors in healthy human fibroblasts^18^. *PINK1* expression was not altered at basal levels (Fig. 3i) and unlike 2 N cells (*p* < 0.01; Fig. 3i), was not efficiently upregulated after CCCP exposure in DS. Although *SQSTM1* expression was significantly increased in both cell lines, the rise was greater in 2 N cells (Fig. 3i).

**Fig. 4:**
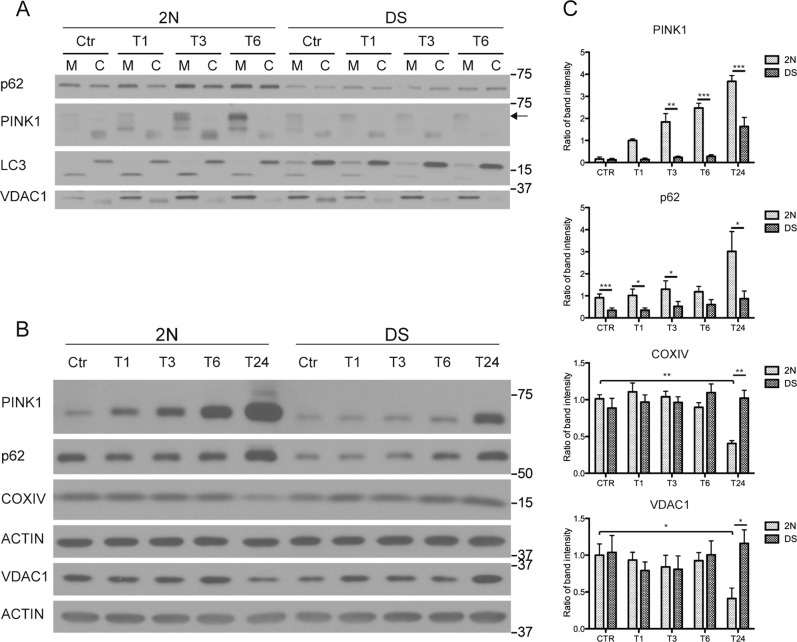
Impairment of mitophagy upon Antimycin A and Oligomycin exposure. **a** Cells were treated with Antimycin A (1 μM) and Oligomycin (1 μM) for 1, 3, and 6 and mitochondrion/cytosol fractionation was performed to assay the accumulation of LC3-II, p62 and PINK1 within the mitochondrial fraction. VDAC1 was used as a loading control for mitochondrial fractions. **b, c** Cells were treated with Antimycin A (1 μM) and Oligomycin (1 μM) for 1, 3, 6, and 24 h and total fibroblasts extracts were analyzed for PINK1, p62, COXIV and VDAC1. Each quantitative data was normalized with ACTIN (*n* ≥ 3). Statistical analysis was performed using two-way ANOVA with Tukey’s multiple comparisons test. (*p < 0.05; **p < 0.01; ***p < 0.001). Immunoblots reported are from one representative experiment

CCCP is commonly used to induce mitophagy, but it can also cause ER stress^49^, and prevent lysosomal acidification^51^. Therefore, we confirmed mitophagy impairment in DS fibroblasts with a milder mitophagy stimulus consisting of a combination of antimycin A and oligomycin (AA/OA)^28^. We monitored AA/OA-induced translocation of p62, LC3-II and PINK1 stabilization in enriched mitochondrial fractions, observing a time-dependent accumulation of PINK1 and p62 within 1 h in 2 N but not in DS cells (Fig. 4a) where neither PINK1 nor p62 significantly changed over 6 hr (Fig. 4a). Of note, AA/OA treatment involves accumulation of p62 in the mitochondrial fraction suggesting mitophagy induced in this manner differs somewhat from CCCP treatment although each revealed a deficit in DS.

Since prolonged mitochondrial depolarization can cause mitochondrial protein depletion due to mitophagy-mediated removal of damaged mitochondria, we examined total cell extracts. Upon extended AA/OA treatment, PINK1 levels in 2 N cells rose within 6 h, but the PINK1 response was delayed in DS cells and remained lowered up to 24 h (*p* < 0.05, Fig. 4b, c) indicating a deficit in DS cells in PINK1/PARKIN-mediated mitophagy upon mitochondrial damage. Levels of the mitochondrial proteins COXIV and VDAC1 decreased only in 2 N fibroblasts accompanied by a time-dependent accumulation of PINK1 (Fig. 4b, c). Collectively, these results indicate defective PINK1/PARKIN-mediated mitophagy in DS fibroblasts, further contributing to cellular accumulation of damaged mitochondria.

### Macroautophagy induction and autophagosome formation are aberrantly down-regulated in DS fibroblasts

**Fig. 5:**
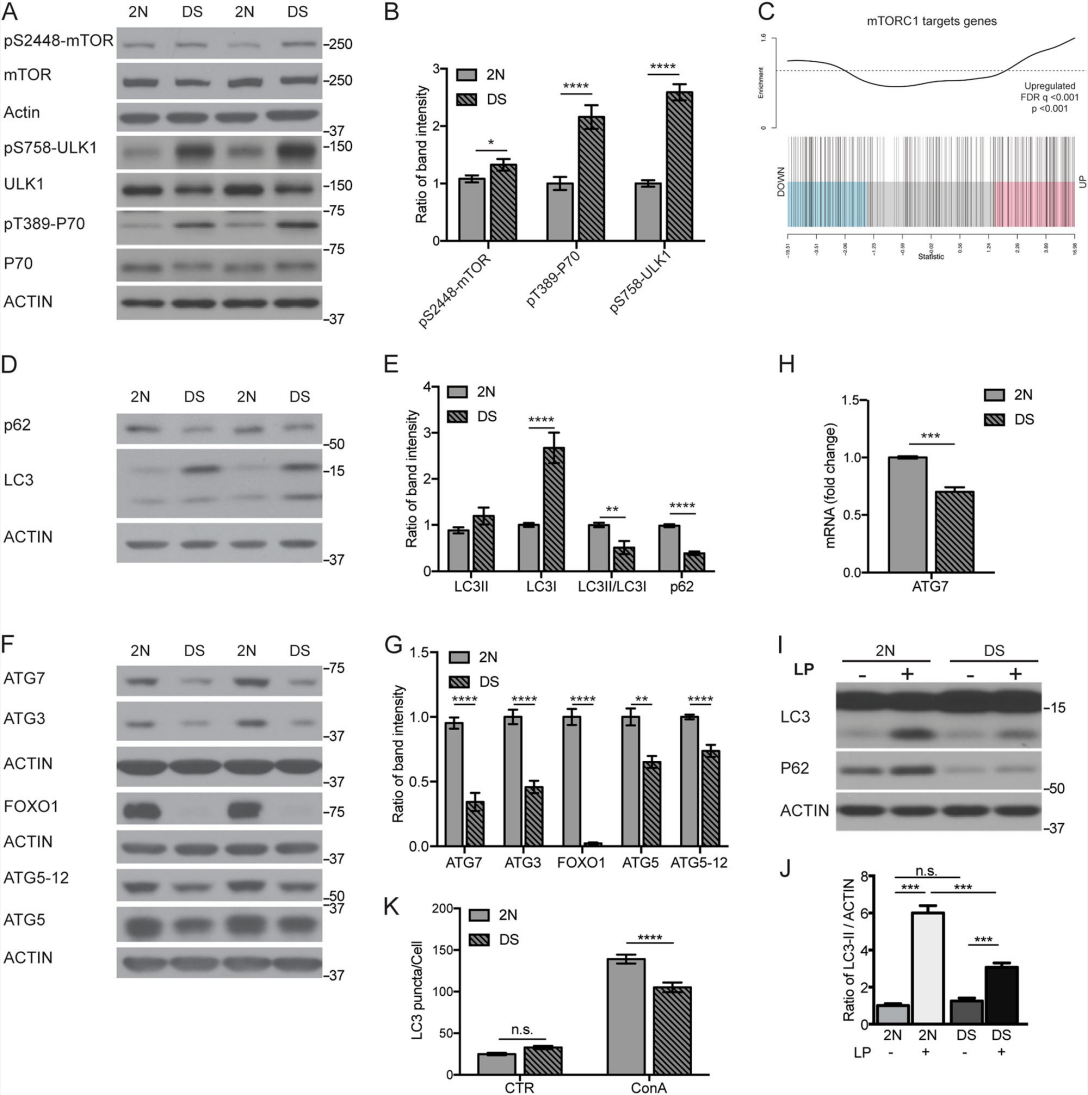
Hyperactivation of mTOR and impairment of autophagy. **a** Total fibroblasts extracts were subjected to SDS-PAGE and analyzed for mTOR, ULK1 and P70S6K (P70), the phospho-specific antibodies were used to determine mTOR activity. **b** Phosphorylation levels quantified by densitometry as a ratio of phospho-protein/total protein level (*n* ≥ 3). **c** GSEA of transcriptome changes comparing DS fibroblasts to 2 N fibroblasts, using a list of genes described to be upregulated upon hyperactivated mTOR signaling due to *Tsc1* depletion (Shin et al. ^34^). As described for Figs. 1d and 2i, the vertical bars represent genes horizontally ranked by moderated t-statistics; Pink, blue and grey shaded rectangles upregulated, downregulated and genes that are unchanged, and the trace above the bar highlights relative enrichment of genes (see Methods sections for other details). The plot shows significant upregulation of this list of genes in DS compared to 2 N fibroblasts. (*p* < 0.01; FDR < 0.01). **d** Whole-cell extracts were analyzed by western blot for p62, LC3-I, LC3-II with ACTIN used as a loading control with quantitation in (**e**). (*n* ≥ 3). **f** Whole-cell extracts were analyzed by western blot for highly relevant autophagic proteins ATG7, ATG3, FOXO1, ATG5 and ATG5-12 complex with ACTIN used as a loading control with quantitation in (**g**). (*n* ≥ 3). **h** 2 N and DS cells were assessed for p62 and AGT7 mRNA by quantitative real-time PCR. mRNA levels were normalized to ACTIN mRNA, used as internal control. Data display the fold-changes of *ATG7* mRNAs relative to control cells (*n* ≥ 3, extracts prepared from independent experiments). **i** Western blot analysis of LC3 and p62 in 2 N and DS fibroblasts treated with vehicle or both Leupeptin and Pepstatin A (LP, 10 μM for 24 h) with quantitation shown in (**j**). (*n* = 4). **k** Quantification of endogenous LC3 puncta structures per cell in the presence or absence of lysosomal inhibitor Concanamycin A (ConA, 50 nM; for 2 h) in DS and 2 N cells. Quantification based upon counts derived from images shown in Fig. 2h. Quantitation based on a minimum of 70 cells per conditions from three independent experiments. All values are shown as the mean ± SEM. Statistical analysis was performed using Student’s *T*-test or two-way ANOVA with Tukey’s multiple comparisons test. (*p < 0.05; **p < 0.01; ***p < 0.001; *n.s*. no significant difference). Immunoblots reported are from one representative experiment

Mitophagy is a form of selective autophagy but still requires the same protein complexes deployed in macroautophagy to form and clear APs^28^. We therefore investigated the molecular cascade regulating autophagy flux starting from the AKT-MTOR signaling pathway. Western blot analysis of DS fibroblasts showed that dual phosphorylation on Thr^308^/Ser^473^ of AKT, yielding maximum activity^52^, was significantly increased (*p* < 0.01; Suppl. Fig. 2a, b). Next, we investigated whether AKT activation also regulated mTORC1^52^. Consistent with this finding, immunoblot analysis demonstrated increased phosphorylation at S^2448^ within the kinase catalytic domain of mTOR in DS cells, indicating abnormally elevated mTOR activity (*p* < 0.01; Fig. 5a, b) as also reported in DS brain and primary cortical neurons from a DS mouse model^25,26,53^. Consistent with mTOR hyperactivation, phosphorylation of P70 ribosomal S6 kinase (P70S6K), a direct substrate of mTORC1^54^, was increased (*p* < 0.001; Fig. 5a, b). Moreover, increased phosphorylation of ULK1-S^758^, reflected activity inhibition and suppression of autophagy induction in DS fibroblasts relative to 2 N controls (*p* < 0.001; Fig. 5a, b). Supporting these findings, we interrogated by GSEA analysis genes known to be targeted by hyperactivated mTOR signaling in TSC1 KO^34^, which revealed an enrichment in DS (*p* < 0.001, FDR *q* < 0.001; Fig. 5c).

Given that the AKT-mTORC1 cascade negatively regulates autophagy^22^, we investigated whether AP formation was also affected in DS fibroblasts. Despite markedly higher levels of LC3-I protein, correlating with significantly increased *MAP1LC3B* mRNA levels in DS cells (Suppl. Fig. 1h), we observed a reduction of LC3-II/LC3-I ratio (−80%, *p* < 0.001; Fig. 5d, e). Accordingly, ATG5 protein, involved with lipidation of LC3 and its family members^55^, was downregulated (−35%, *p* < 0.001) (Fig. 5f, g) and in the ATG12–ATG5 conjugated complex (Fig. 5f, g). Moreover, p62 protein (*p* < 0.001; Fig. 5d, e) and *SQSTM1* expression (*p* < 0.001; Fig. 2c and 3i) were also lowered. Finally, proteins critical for AP elongation, ATG7, a E1-like enzyme, and ATG3, a E2-like enzyme^55^, were markedly downregulated at both the protein (*p* < 0.001; Fig. 5f, g) and mRNA levels (RNA-seq data, Suppl. Fig. 2c; qPCR for *ATG7*, *p* < 0.001; Fig. 5h).

Although these observations strongly suggested that autophagy induction was downregulated in DS fibroblasts, we confirmed this directly by measuring AP formation in presence of leupeptin-pepstatin A (LP) to prevent AP clearance. After 24 h of treatment, LC3-II was lower in DS cells (*p* < 0.001; Fig. 5i, j). As previously shown, impaired lysosomal degradation of autophagic substrates^45^ causes an abnormally low rate of clearance of LC3-II-positive APs and thus, basal LC3-II levels were not significantly lowered in DS cells compared to 2 N cells (Fig. 5e, j). Quantification of LC3 puncta number/cell further verified the autophagy flux impairment in DS cells with a marked decrease of LC3 puncta number compared to 2 N in the presence of ConA (*p* < 0.0001; Fig. 5k). Collectively, these data show that AP formation and maturation in DS is downregulated at multiple levels: from depressed induction (increased AKT-mTORC1 axis signaling) to lowered expression of key ATG genes required for AP formation shown herein, and likely additional deficits of lysosomal clearance^45^.

### Inhibition of mTOR rescues mitophagy defects and macroautophagy suppression in DS fibroblasts

**Fig. 6:**
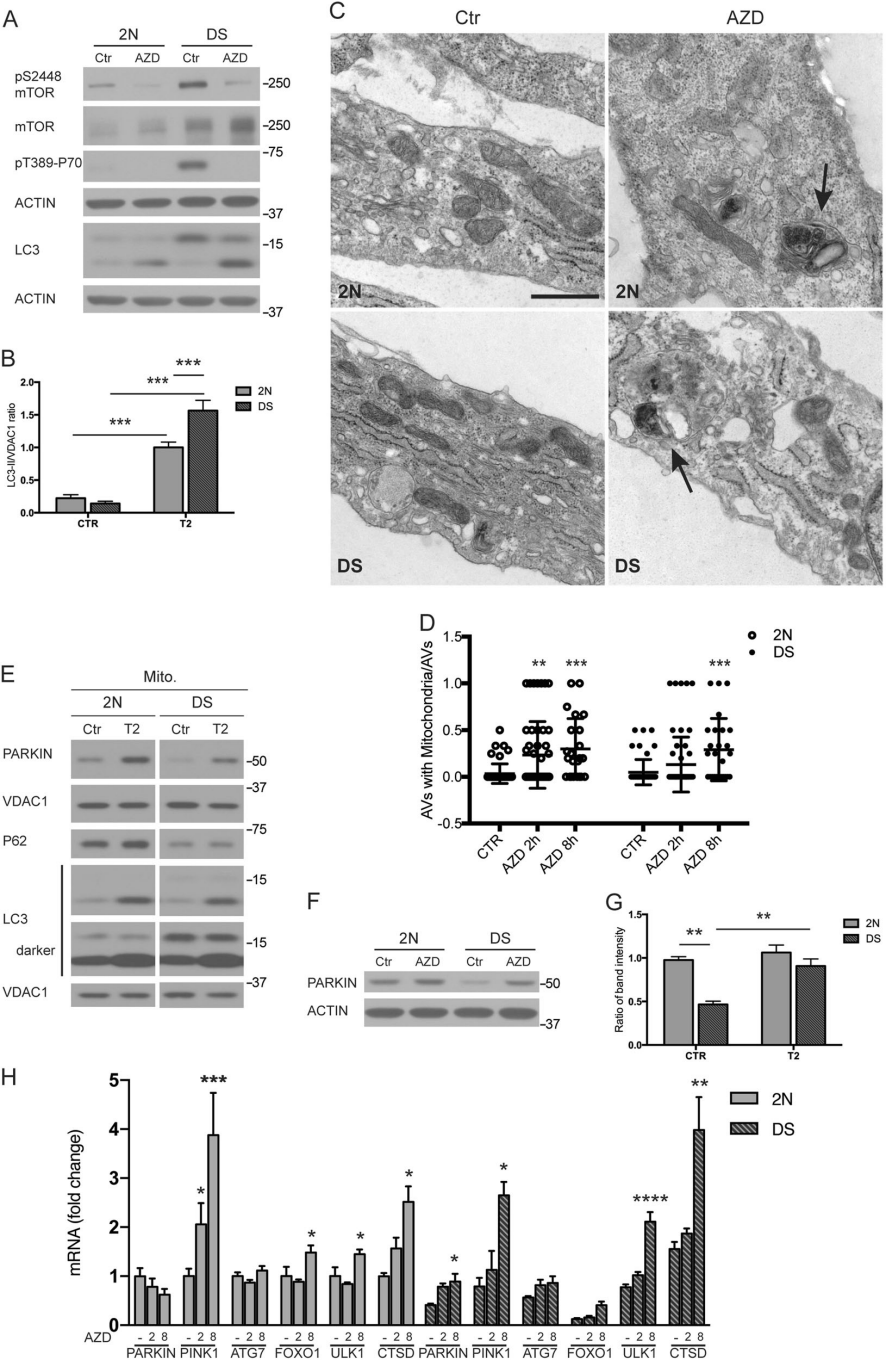
Rescue of autophagy and mitophagy induction defects in DS by mTOR inhibition. **a** 2 N and DS fibroblasts were treated with AZD8055 (AZD; 0.1 μM), an mTOR specific inhibitor, for 2 h and whole-cell extracts were assessed for p62, LC3, P70S6K (P70) and mTOR; the phospho-specific antibodies were used to determine mTOR activity. **b** LC3-II levels following AZD treatment quantified and normalized with ACTIN (*n* = 4). **c** Electron microscopy analysis of 2 N and DS cells after 2 h AZD treatment. Black arrows point to autophagosome/autolysosomes with engulfed mitochondria. Scale bar 500 nm. **d** Quantification of autophagosome/autolysosomes (autophagic vacuoles, AVs) with engulfed mitochondria after 2 and 8 h AZD treatment, graph shows the ratio AVs with Mitochondria/AVs total number. **e** The cells were treated with AZD (0.1 μM) for 2 h and mitochondrion/cytosol fractionation was performed to assay the accumulation of PARKIN, p62, ATG7 and LC3-II within the mitochondrial fractions. VDAC1 was used as a loading control. **f, g** Western evaluation of PARKIN levels upon 2 h AZD treatment and relative quantification normalized with ACTIN. **h**. 2 N and DS cells were treated with AZD for 2 h and 8 h and were assessed for *PARKIN* and *PINK1*, ATG7*, FOXO1, ULK1*, and *CTSD* (Cathepsin D) mRNA by quantitative real-time PCR. mRNA levels were normalized to *ACTIN* mRNA, used as internal control. Data display the fold-changes of gene expression relative to control cells (*n* ≥ 3). Statistical analysis was performed using one-way or two-way ANOVA with Tukey’s multiple comparisons test for Real Time PCR. Immunoblots reported are from one representative experiment. (*p < 0.05; **p < 0.01; ***p < 0.001)

To further investigate the relationships of altered PINK1/PARKIN dependent mitophagy and down-regulated autophagy induction to accumulation of damaged mitochondria, we inhibited mTORC1 activity with AZD8055 (AZD) to promote autophagy and possibly mitophagy. As expected, AZD diminished mTORC1 activity within 2 h as evidenced by decreased p-P70 and p-mTOR (Fig. 6a). At this time point, LC3-II levels rose in both cell lines (Fig. 6a, b) but to a greater extent in DS likely due to impaired lysosomal degradation (Fig. 5e, j), previously established in these cells^45^. To assess AZD effects on mitophagy, we analyzed EM images to detect mitochondria sequestered within APs. APs containing mitochondria were detected in both cell lines (Fig. 6c, arrows), implying that mTOR inhibition promotes mitophagy. The numbers of AP/AL containing mitochondria significantly increased after 2 h of treatment in 2 N cells and after 8 h in DS cells (Fig. 6d). Western blot analysis confirmed this result, showing a rise of mitochondrial LC3-II level and PARKIN translocation in mitochondrial fractions upon 2 h of AZD (Fig. 6e) in both DS and 2 N cells. Moreover, at this time point, we detected a significant upregulation of PARKIN total protein levels compared to basal condition in DS cells (Fig. 6f, g).

We evaluated whether mTOR inhibition through AZD could reverse the downregulated expression in DS cells of components involved in AP formation (e.g., ATG7 and FOXO1) and mitophagy (PARKIN) found herein. Reports revealed how mTORC1 negatively regulates autophagy also at the transcriptional level by inhibiting, for example, the members of microphthalmia/transcription factor E (MiT/TFE) subfamily (such as TFEB and TFE3)^56,57^. In fact, upon mTOR inhibition (starvation or ATP-competitive mTOR inhibitors), TFEB and TFE3 translocate into the nucleus to promote expression of target genes supporting the activation of autophagy and lysosomal biogenesis^56,57^. qPCR analysis revealed AZD treatment induced elevation in *PINK1*, *ULK1* and *CTSD* (Cathepsin D) expression in both cell lines (Fig. 6h) in a time-dependent manner. Intriguingly, in DS cells, AZD significantly upregulated *PARKIN* and there was a trend toward increases for *ATG7* and *FOXO1*, which were downregulated in untreated DS cells (*p* ≤ 0.01, Fig. 6h). These results demonstrate rescue of both autophagy and PINK1/PARKIN related mitophagy deficits in DS cells when autophagy induction is stimulated.

**Fig. 7:**
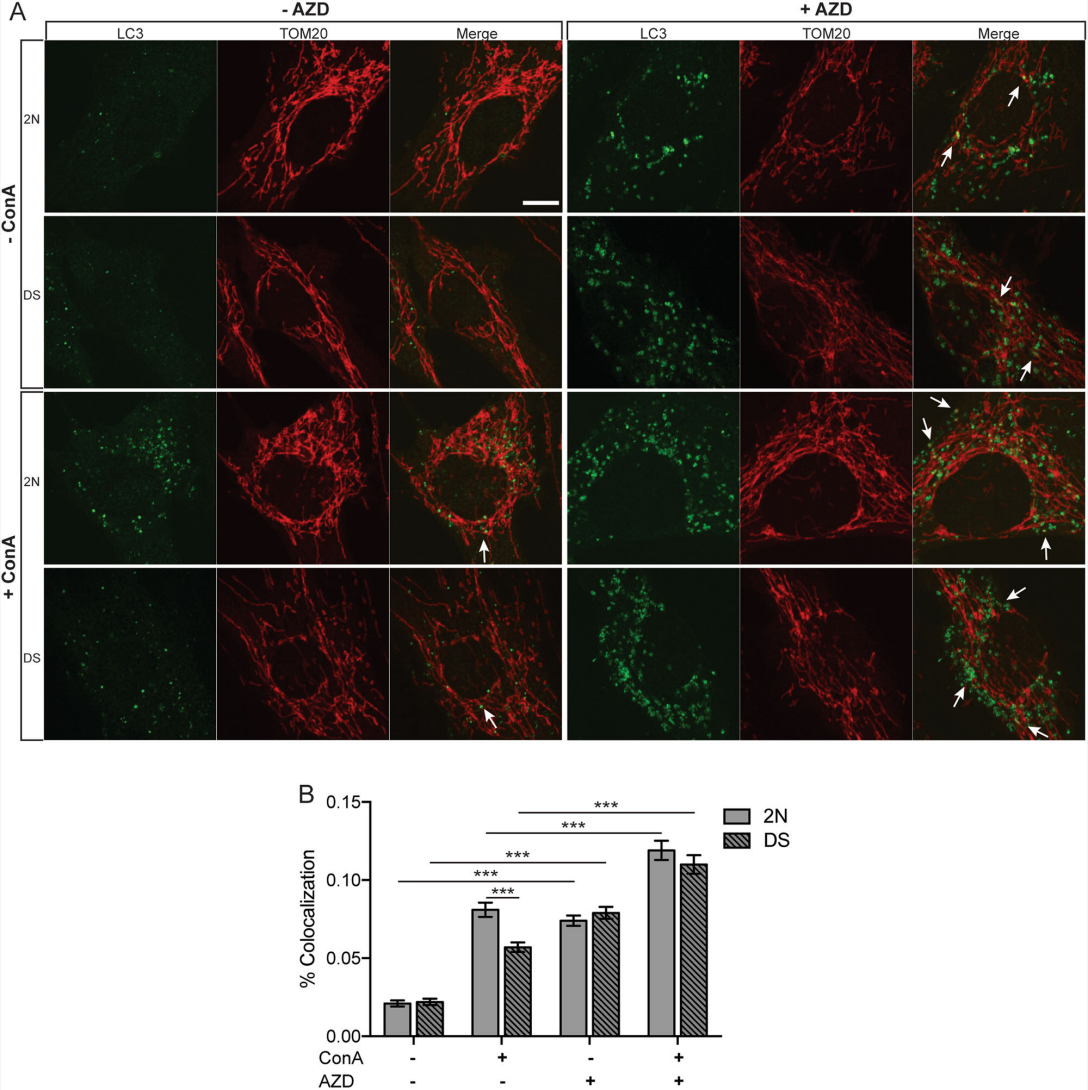
AZD8055 promotes colocalization of mitochondria with autophagosomes. **a** 2 N and DS Fibroblasts were treated with AZD (0.1 μM), with ConA (50 nM) or both compounds together for 2 h. The cells were fixed and immunostained for LC3 and TOM20 as shown. **b** The graph shows the percentage of LC3/Tom20 colocalization calculated by JACoP plugin of ImageJ. A minimum of 80 cells were counted per condition from three independent experiments. White arrows point to the co-localization of signals in puncta. Statistical analysis was performed using one-way ANOVA with Tukey’s multiple comparisons test. Scale bar 10 μm (*p < 0.05; **p < 0.01; ***p < 0.001)

Finally, we repeated the immunostaining for endogenous LC3 and TOM20 upon AZD treatment with and without ConA (Fig. 7). Following 2 h treatment, the colocalization of LC3^+^ puncta with TOM20 increased significantly over baseline, with further enhancement after ConA (*p* ≤ 0.001, Fig. 7b). Notably, the combination treatment suppressed the differences between the two cell lines observed in presence of ConA alone. Collectively, our findings demonstrate that mTOR inhibition, which restores macroautophagy and mitophagy activity in DS cells, rescues the abnormal accumulation of damaged mitochondria.

## Discussion

**Fig. 8:**
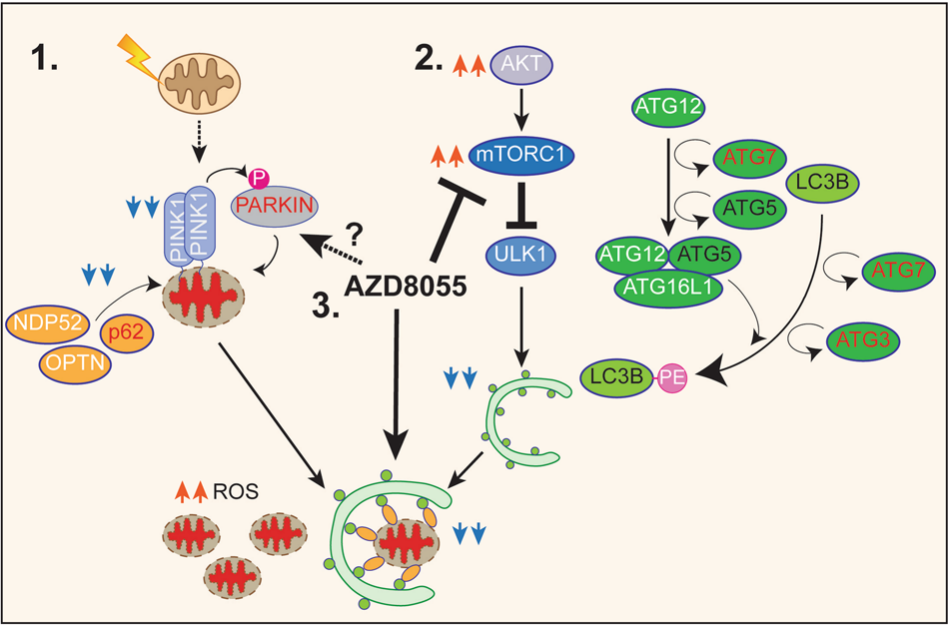
Proposed model illustrating the autophagy and mitophagy defects in Down Syndrome fibroblasts. Our findings indicate that (**a**) PARKIN/PINK1-mediated mitophagy pathway is altered in DS human fibroblasts leading to an accumulation of damaged mitochondria and thus to increase of ROS levels. We also find that macroautophagy is negatively regulated at different levels (**b**) mTOR is hyperactivated leading to an inhibition of autophagy induction and crucial proteins involved in the autophagosome formation, are down-regulated, including ATG7 and FOXO1. **c** AZD8055 (AZD), an inhibitor of both of mTOR complex 1 (TORC1) and complex 2 (TORC2), rescues mitophagy and autophagy flux, thereby promoting the clearance of damaged mitochondria. The extensive autophagic deficits likely contribute in different ways to the severe symptoms described in DS individuals, leading to the supposition that TORC1-TORC2 inhibition can be a promising therapeutic option for Down Syndrome. Factors downregulated in DS cells highlighted as follows: titles in red denote those downregulated at both the mRNA and protein level, black downregulated at the protein level, white are those that are unchanged. Blue arrowheads indicate those pathways downregulated while orange arrowheads those upregulated. See text for further details

We used a multi-faceted approach to comprehensively analyze mitophagy and autophagy deficits in DS fibroblasts. While confirming significant accumulation of damaged mitochondria associated with increased oxidative stress^7–9,37^, we found that factors involved in transcriptional control of mitochondrial biogenesis and metabolism, including PGC-1α^7^, were lowered. Despite the possibility that of lowered mitochondrial biogenesis, total mitochondrial mass was increased, raising suspicions that deficient mitophagy could explain increased numbers of damaged mitochondria and total mitochondrial mass. Consistent with this possibility, we established multiple lines of evidence (Fig. 8) revealing two sources of mitophagy suppression in DS cells: firstly, downregulated PARKIN associated with perturbed PINK1-generated mitophagy signaling and secondly, decreased mitophagy activity from downregulated mTOR-dependent macroautophagy. Notably, both impairments of mitophagy were rescued by blocking mTOR with AZD, suggesting the therapeutic potential of this class of autophagy enhancers.

Mitophagy is a specialized type of autophagy but uses core machinery similar to non-selective macroautophagy to support sequestration and clearance steps. In fact, activation of the PINK1-PARKIN axis recruits on mitochondria proteins mediating AP biogenesis including ULK1, DFCP1 and WIPI1^17,55^. ULK1 activation coordinates mitophagy^58^ and is required to further recruit downstream ATG proteins^59^. Accordingly, *Ulk1* knockout mice exhibit mitophagy defects and accumulation of aberrant mitochondria^60^. We also found that mTORC1 hyperactivation in DS fibroblasts catalyzed the inactivating phosphorylation of ULK1, resulting in decreased autophagy induction and potentially a decline in mitophagy. The AKT-mTORC1 axis was also shown to be hyperactivated in DS cells, as supported by transcriptomic analysis, which corroborates and extends findings in DS human brain and primary cortical neurons from the DS mouse model Ts1Cje^25,26,53^. Although the basis for the hyperactivated AKT-mTORC1 cascade in DS cells is not yet identified and may be multifactorial, mTORC1 hyperactivation accounts for the strong suppression of autophagy, and as our AZD findings indicate, of mitophagy.

We identified components of two ubiquitin-like conjugation systems critical for AP initiation and maturation^28^ that were downregulated, including ATG7, ATG3, FOXO1 (as direct ATG7 interactor)^61^, ATG5 and ATG5-12 complex. These findings support down-regulation of autophagy induction at multiple levels and not only from lowered ULK1 activity due to mTORC1-mediated S^758^ phosphorylation. Furthermore, increased oxidative stress in DS cells resulting from diminished elimination of dysfunctional mitochondria, may further compromise the autophagy-mitophagy process, thereby establishing a negative regulatory feedback loop. For example, high ROS generation alters PARKIN function and can disrupt its E3 ligase activity^62,63^ and compromise mitophagy. More recently, Frudd and collaborators have shown that oxidative stress inhibits autophagy by directly oxidizing ATG7 and ATG3^64^, which we also find downregulated in trisomic fibroblasts.

mTORC1 negatively regulates autophagy in multiple ways, including blocking ULK1 activity and preventing nuclear translocation of TFs supporting AP and lysosomal biogenesis^54,65,66^. Importantly, we established that mTOR inhibition by AZD8055^27^ both reversed autophagy induction deficits in DS cells within 2 h of treatment and restored mitophagy levels in DS fibroblasts. Moreover, AZD restored expression of autophagic and mitophagic genes downregulated in untreated DS including *ATG7*, *FOXO1* and *PARKIN* while significantly elevated *PINK1* mRNA levels, thus underscoring its positive effect on mitophagy induction. The beneficial effects of AZD on DS fibroblasts at multiple levels may be related to its inhibitory effect on both mTOR complexes. AZD blocks mTORC2 which negatively regulates AKT and activates FOXO1 and FOXO3^27^ which play critical roles in regulating autophagic gene expression^67,68^ and control *PINK1* transcription after growth factor deprivation^69^ or diabetes-induced mitophagy^70^. These observations could explain the positive effects we observed on *PINK1* expression upon AZD treatment.

In conclusion, we have extensively characterized multifactorial alterations of autophagy and mitophagy pathways in DS fibroblasts, providing novel insights into the molecular pathogenesis of Down syndrome. The restoration of autophagy and mitophagy functions with AZD treatment reveals common sites of regulation, suggesting promising approaches to ameliorating deficits associated with Down syndrome in various organs, including brain.

## Supplementary information

Supplementary Table 1

Supplementary Figure S1

Supplementary Figure S2

Supplementary figure legends
