# Supplementary material for: mTOR hyperactivation in Down Syndrome underlies deficits in autophagy induction, autophagosome formation, and mitophagy
Source: Cell Death Dis. 2019 Jul 22;10(8):563. doi: 10.1038/s41419-019-1752-5 (PMC6646359; doi:10.1038/s41419-019-1752-5)
Supplement: Supplementary file 1 — Supplementary Table 1 [file 41419_2019_1752_MOESM1_ESM.docx]

| **Mitochondrial Transcriptional factors** | **Transcriptional factors to be involved in mitophagy** |
| --- | --- |
| FOXO1 | SIRT2 |
| PPARG | SIRT3 |
| PPARD | SIRT5 |
| SIRT1 | SOX2 |
| STAT3 | MITF |
| PPARGC1A | TFE3 |
| TFB1M | TFEB |
| PHB2 | TFEC |
| TFAM | SREBF1 |
| TFB2M | SREBF2 |
| ACACA |  |
| YY1 |  |
| NFE2L2 |  |
| FOXO3B |  |
| PPARA |  |
| PPID |  |
| ESRRA |  |
| FOXO3 |  |
| FOXO4 |  |
| NRF1 |  |
| ZNF746 |  |
