# Supplementary figures and images for: mTOR hyperactivation in Down Syndrome underlies deficits in autophagy induction, autophagosome formation, and mitophagy
Source: Cell Death Dis. 2019 Jul 22;10(8):563. doi: 10.1038/s41419-019-1752-5 (PMC6646359; doi:10.1038/s41419-019-1752-5)

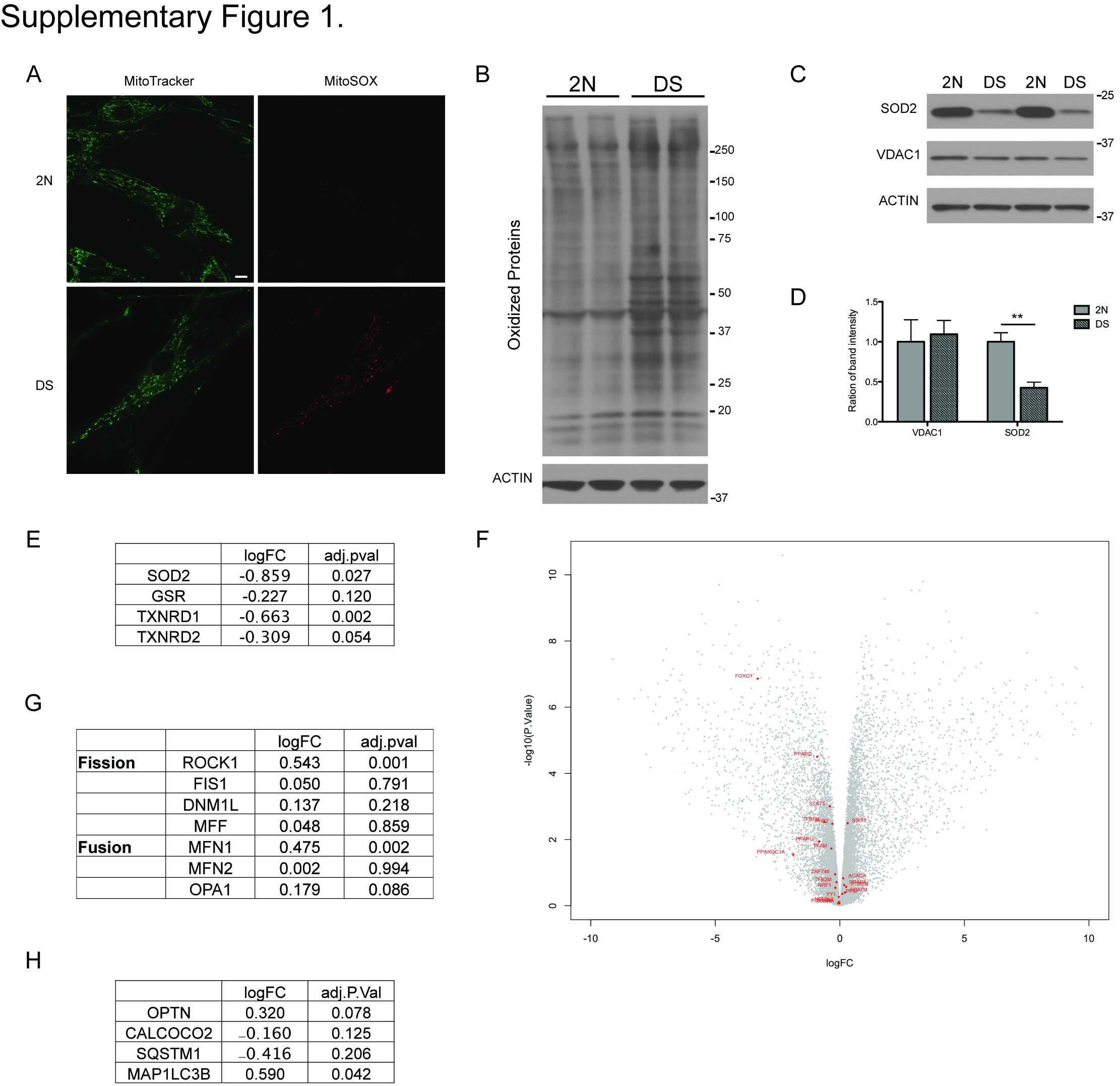

Supplement: Supplementary file 2 — Supplementary Figure S1 [file 41419_2019_1752_MOESM2_ESM.jpg]

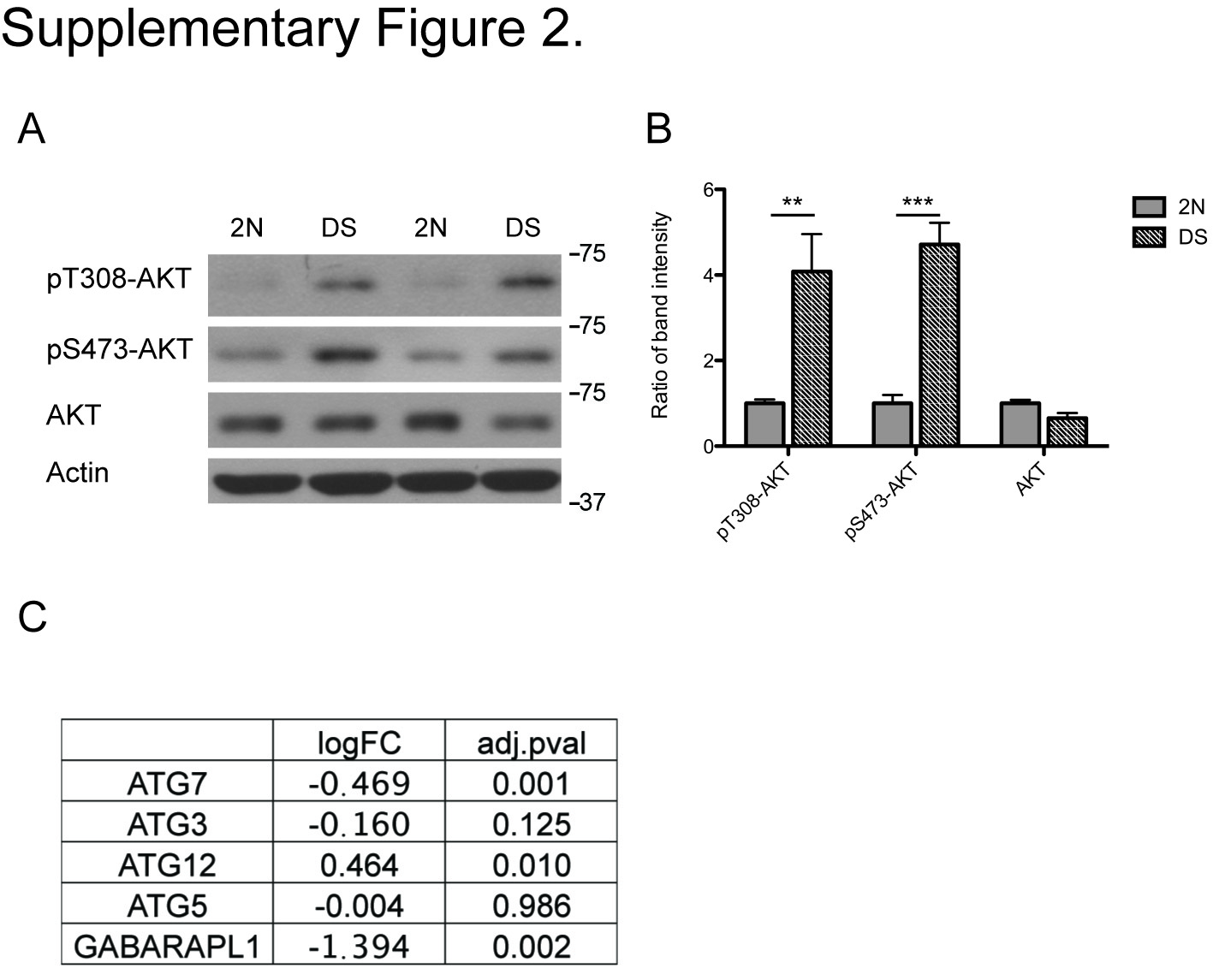

Supplement: Supplementary file 3 — Supplementary Figure S2 [file 41419_2019_1752_MOESM3_ESM.jpg]
