# Supplementary material for: mTOR hyperactivation in Down Syndrome underlies deficits in autophagy induction, autophagosome formation, and mitophagy
Source: Cell Death Dis. 2019 Jul 22;10(8):563. doi: 10.1038/s41419-019-1752-5 (PMC6646359; doi:10.1038/s41419-019-1752-5)
Supplement: Supplementary file 4 — Supplementary figure legends [file 41419_2019_1752_MOESM4_ESM.docx]

**Supplementary Figure 1. Mitochondrial damage, oxidative stress and expression profile of fusion-fission and mitochondrial transcriptional factors in DS fibroblasts**

**A.** Live-scanning confocal microscopy of cells stained with MitoSOXTM (mitochondria-selective superoxide anion indicator) and co-stained with the MitoTracker green (mitochondrial marker); quantification shown in Figure 1B. Scale bar 10μm. **B.** Representative immunoblot showing oxidative damage, revealed by carbonylation of proteins prepared from Whole-cell extracts of 2N and DS cells. **C-D.** Total fibroblasts extracts were subjected to SDS-PAGE and analyzed for SOD2 and VDAC1 (n = 3); each quantitative data was normalized with ACTIN. Statistical analysis was performed using Student’s T-test. (**p<0.01). **E.** Graph showing gene expression changes derived from DS to 2N fibroblasts comparison of antioxidant enzymes Superoxide Dismutase 2 (*SOD2*) and Thioredoxin Reductase-1 and 2 (*TXNRD1* and *TXNRD2*). (Expressed as log2-fold change) **F.** Graph showing genes expression changes of transcriptional factors regulating mitochondrial biogenesis used for GSAA analysis in Figure 1D (expressed as log2-fold change). **G.** Graph showing genes expression changes derived from DS to 2N fibroblasts comparison of most relevant factors involved in fusion and fission pathways. (*p<0.05;**p<0.01;***p<0.001)

**Supplementary Figure 2. Hyperactivation of AKT and alteration of some relevant autophagic genes**

**A.** Total fibroblast extracts were subjected to SDS-PAGE and analyzed for AKT and the phospho-specific antibodies were used to determine AKT activity, pT^308^- and pS^473^. **B.** Phospho levels quantified by densitometry as a ratio of phospho-protein/total protein levels, while AKT was normalized with ACTIN (n ≥ 3). Statistical analysis was performed using Student’s T-test. **C.** Graph showing genes expression changes derived from DS to 2N fibroblasts comparison of *ATG7*, *ATG3*, *ATG12*, *ATG5*, *MAP1LC3B*, *GABARAPL1* and *SQSTM1* (expressed as log2-fold change). (*p<0.05;**p<0.01;***p<0.001)

**Supplementary Table 1.**

List of transcriptional factors regulating mitochondrial biogenesis^7,42,43^ and of transcriptional factors that have been described to be involved in mitophagy^46,47^ used for GSEA analysis in Figure 1D and 2I, respectively.
